# Rumor Diffusion and Convergence during the 3.11 Earthquake: A Twitter Case Study

**DOI:** 10.1371/journal.pone.0121443

**Published:** 2015-04-01

**Authors:** Misako Takayasu, Kazuya Sato, Yukie Sano, Kenta Yamada, Wataru Miura, Hideki Takayasu

**Affiliations:** 1 Interdisciplinary Graduate School of Science and Engineering, Tokyo Institute of Technology, Yokohama, Kanagawa, Japan; 2 College of Science and Technology, Nihon University, Funabashi, Chiba, Japan; 3 Faculty of Engineering, Information and Systems, University of Tsukuba, Tsukuba, Ibaraki, Japan; 4 Waseda Institute for Advanced Study, Shinjuku, Tokyo, Japan; 5 Graduate School of Engineering, The University of Tokyo, Tokyo, Japan; 6 PRESTO, Japan Science and Technology Agency, Kawaguchi, Saitama, Japan; 7 Sony Computer Science Laboratories, Inc., Shinagawa, Tokyo, Japan; 8 Meiji Institute for Advanced Study of Mathematical Sciences, Kawasaki, Kanagawa, Japan; Centrum Wiskunde & Informatica (CWI) & Netherlands Institute for Systems Biology, NETHERLANDS

## Abstract

We focus on Internet rumors and present an empirical analysis and simulation results of their diffusion and convergence during emergencies. In particular, we study one rumor that appeared in the immediate aftermath of the Great East Japan Earthquake on March 11, 2011, which later turned out to be misinformation. By investigating whole Japanese tweets that were sent one week after the quake, we show that one correction tweet, which originated from a city hall account, diffused enormously. We also demonstrate a stochastic agent-based model, which is inspired by contagion model of epidemics SIR, can reproduce observed rumor dynamics. Our model can estimate the rumor infection rate as well as the number of people who still believe in the rumor that cannot be observed directly. For applications, rumor diffusion sizes can be estimated in various scenarios by combining our model with the real data.

## Introduction

Online social media have become ubiquitous since the beginning of this century. People use social media in both everyday life and emergencies. As a result, analysis of large amounts of social media data has become the target of researchers for both application-oriented topics and basic scientific studies. For instance, Google flu trends (http://www.google.org/flutrends/) can report influenza epidemics in any area in real time [1]. A combination of GPS information and texts on social media, such as geotagged tweets, can predict the probability of infection on an individual basis [2], which allows a novel perspective of public health. It is also possible to predict movie box-office revenue [3], to estimate macroeconomic statistics such as unemployment rates [4], and to scientifically study online retailers’ sales rank behavior as a typical case of ranking fluctuations [5]. Furthermore, with the growing number of people using online social media, politicians can no longer ignore their opinions [6–9].

The ease and convenience of social media mean that online rumors diffuse more rapidly and widely than their conventional counterparts. For instance, the retweet function on Twitter enables a subscriber to forward copies of a received tweet to their followers with a single click, allowing tweets to diffuse quickly to a substantial number of people. In 2013, the World Economic Forum described online rumors as “digital wildfire,” and highlighted their risk [10, 11]. Like real wildfires, digital wildfires spread rapidly and can become uncontrollable, even if the information is false or scientifically unfounded.

Since ancient Roman times, there have been reports of rumors that diffused in emergency situations, such as natural disasters and wars [12, 13]. As recently as 2012, rumors diffused when hurricane Sandy hit the north-eastern United States. For example, there were rumors that the government was issuing food stamps for free and that the New York stock exchange was underwater. In response to this problem, the U.S. government established a special section for “rumor control” on the Federal Emergency Management Agency website (http://www.fema.gov/hurricane-sandy-rumor-control) to correct the rumors. However, it is not just misinformation that spreads through social media. Useful information can also appear on social media during an emergency situation, the study of which is a growing area of research [14–16].

Rumor diffusion, or information cascade, is associated with phase-transition phenomena showing sudden qualitative changes under continuous variation of parameters, and has been attracting attention from scientists [17–26]. Mathematical models of rumor diffusion have been analyzed for many types of networks, such as random networks [20, 21] and scale-free networks [22–24].

In this study, we examine real diffusion and convergence process of one particular rumor by analyzing all Twitter data written in Japanese during the week beginning March 11, 2011, the day of the Great East Japan Earthquake (the 3.11 Earthquake). We first show an overview of a rumor diffusion, then we explain how a correction tweet sent from a city hall account diffused enormously by retweets. We introduce a mathematical model of rumor diffusion inspired by an agent-based stochastic contagion model of epidemics, widely known as the Susceptible-Infected-Recovered (SIR) model, and show that our model presents an accurate picture of rumor diffusion dynamics by combining our model with the real data.

## Results

### Rumor Diffusion

An earthquake registering 9.0 on the Richter scale hit the east coast of Japan at 14:46 (JST) on March 11, 2011, followed by many aftershocks. People rushed to the Internet in search of information that had not yet been broadcast and to send messages to relatives and friends, as many telephone lines were destroyed or jammed. Electricity and Internet connectivity resumed in the Tokyo metropolitan area and western Japan, allowing many people to access social media such as Twitter, the most popular social platform [27]. Most online information was helpful, but false or unfounded information also appeared.

The abnormal sequence of aftershocks caused a liquefied petroleum gas (LPG) tank to fall at 15:15 in an oil company located on the eastern shore of Tokyo Bay, and the leaked gas ignited. People living near the gas tank uploaded photos of the fire to Twitter soon after the accident, along with worried comments. A series of gas tank explosions began at 17:04 [28] and the number of tweets increased, many expressing fear.

**Fig 1:**
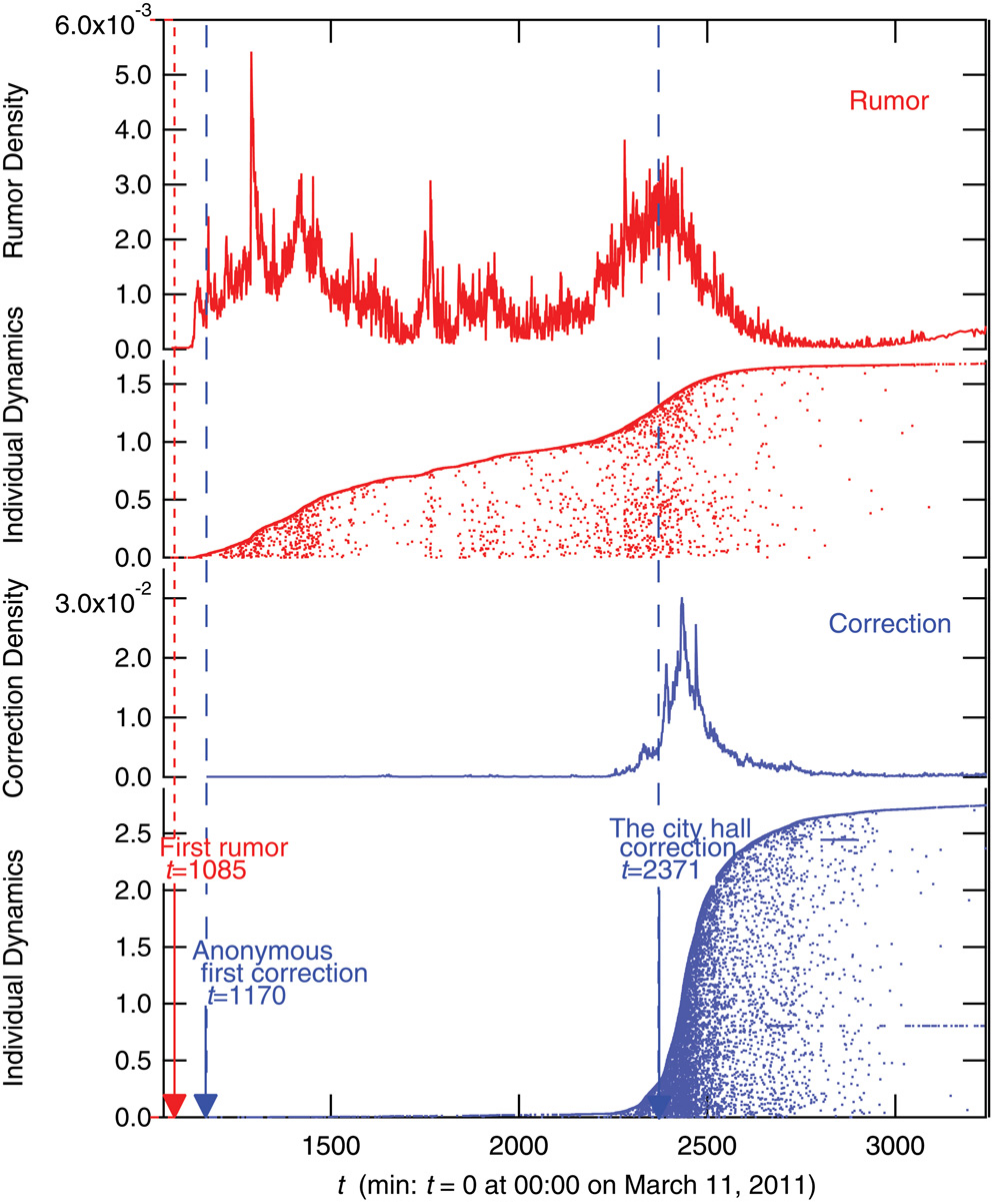
Time evolution and individual dynamics of rumor tweet *R*(*t*) (red) and rumor-correction *R*_*c*_(*t*) tweets (blue). Time series is per minute and normalized by the total number of tweets. In the plot of individual dynamics, the vertical axis is user index, which is ordered by the time of the first appearance in the data set and normalized by the entire tweet volume.

At around 18:00 the first rumor appeared: “Please spread: To those people who live close to the east shore of Tokyo Bay! Due to the explosion of oil tanks, harmful chemical materials may fall with rain soon. Bring your umbrella and rain coat with you to protect your skin from the dangerous rain!!” This warning turned out to be false information with no scientific basis, but many people spread the rumor via Twitter, as shown in Fig. 1. “Individual Dynamics” in Fig. 1 shows the temporal activity of individual users ordered by the time of the first tweet in the dataset on the vertical axis. The numbers on the vertical axis are normalized by the whole number of tweets and the scale on the horizontal axis is measured per minute. Successive dots for the same horizontal direction mean that the same user has tweeted continuously.

The false information was transmitted by a Twitter user who had roughly 360 followers in our data. However, in the end, a total of 88,657 users were eventually involved in the rumor diffusion (38,226 users) and rumor correction (56,818 users). Note that we cannot identify the only one user who actually started the rumor, because some users who tweeted the rumor have already deleted their tweets, and they do not appear in our data. Further, the same rumor emerged almost simultaneously from different users.

### Rumor Convergence

By observing the tweet data in detail, we found that the first correction tweet, which mentions clearly that the rumor tweets about the chemically contaminated rain were scientifically false, was submitted at 19:30 on March 11, just 90 minutes after the appearance of the first rumor tweet. However, at this stage, we found that the number of rumor tweets grew much faster than the number of rumor-correction tweets, as shown in Fig. 1. Obviously, the correction tweet could not stop the diffusion of the rumor in the early stages.

The blue line in Fig. 1 shows that the number of correction tweets increased enormously around 15:00 on March 12 (*t* = 2340), about 21 hours after the first rumor tweet. This corresponds to the time that an official home page of the City Hall of Urayasu, located near the exploded gas tanks, announced that the rumor of chemically contaminated rain was not scientifically valid. In addition, the City Hall sent correction tweets directly to around 15,000 followers, stating “After the LPG tanks explosion, there are rumors that harmful chemically contaminated rain may fall. However, the Earthquake Disaster Prevention Division of the City Fire Department confirmed that there is no scientific basis for these rumors. Please be careful not to be confused by the rumors.” After this municipal action, the rumor-correction tweets spread quickly, and the rumor tweets soon disappeared.

**Fig 2:**
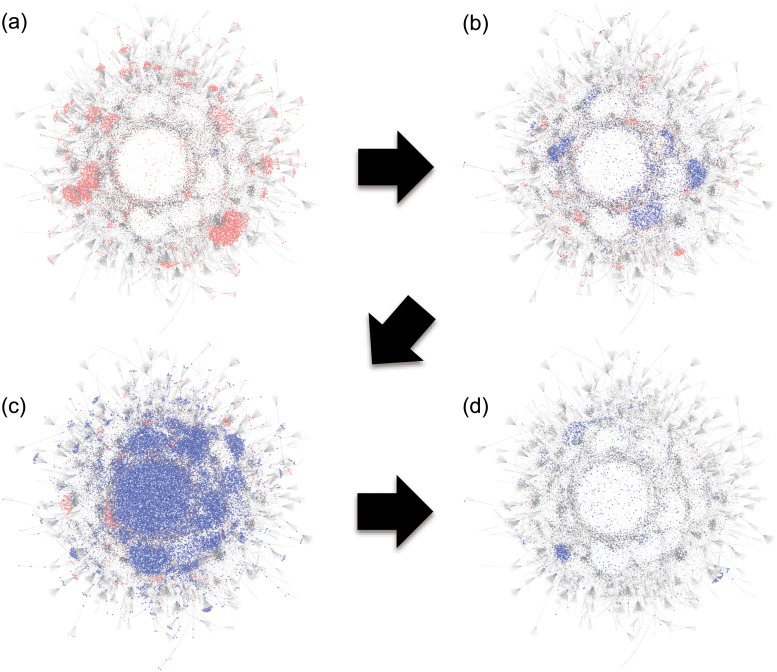
Snapshots of retweet network in three-hour time windows. Each node represents an active user and a link shows a retweet. (a) The rumor (red) is well diffused at 21:00–23:59 on March 11. (b) The rumor and correction (blue) coexist at 12:29–15:30 on March 12. (c) The correction spreads quickly soon after the City Hall tweet at 15:31–18:30 on March 12. (d) The rumor converges at 21:00-23:59 on March 12.

Fig. 2 shows the time evolution of the retweet networks about the rumor tweets and correction tweets. Here, each node represents a Twitter user and each link represents at least one retweet between users. At 21:00 on March 11 (Fig. 2(a)), three hours after the first rumor, there were many rumor tweets (shown by red dots) and almost no correction tweets (blue dots). About 15 hours later (Fig. 2(b)), the numbers of rumor tweets and correction tweets were about the same. Soon after the official correction tweets from the City Hall (Fig. 2(c)), the correction tweets overwhelmed the rumors and the rumors disappeared (Fig. 2(d)).

The largest number of rumor-correction retweets was 21,204, originating from the City Hall. The second largest was 3,402 by the official information center for the earthquake, while the largest number of rumor retweets was 2,078. The final number of nodes in the rumor-correction retweet network is 53,071. This suggests that approximately half of the nodes in the rumor-correction network are linked directly to the City Hall. Furthermore, not only directly retweeted users, but also 37.3% of rumor-correction tweeted users mentioned the City Hall’s tweet. Thus, the City Hall’s correction tweet is outstanding in this case.

There are also several cases in which government officials or public announcements play key roles in stopping rumors. For example, during the aftermath of the 1923 Great Kanto earthquake, the Tokyo Metropolitan Police Department distributed flyers saying that punishment would be meted out to those who told false rumors [29]. An official announcement from the Ministry of Finance and the Bank of Japan stopped a bank run caused by a rumor that led to the withdrawal of 8.7 million dollars during one week in 1973 [30]. Recently, in 2012, the U.S. government corrected rumors during Hurricane Sandy, on the Federal Emergency Management Agency website. In India, there is a law to crack down on rumors that might cause panic during disasters [13]. The Chinese government launched an anti-Internet rumor website (http://py.qianlong.com/) with the cooperation of executives from major Internet companies in 2013. Our results and these pieces of evidence reflect the possibility that official institutions can play active roles in rumor convergence.

### Rumor Simulation

To understand the macroscopic dynamics of rumor diffusion and convergence from the perspective of microscopic Twitter users’ interactions, we simulated rumor diffusion and convergence based on stochastic SIR(susceptible-infected-recovered) model [31], which is one of the well-known minimal models of epidemics such as the influenza virus and Ebola, and reproduces dynamics such as the exponential growth of infected people. Historically, deterministic and stochastic SIR models have been proposed. The deterministic model describes dynamics of population for each state(susceptible-infected-recovered) by ordinary differential equations. On the other hand, the stochastic one describes the dynamics of each individual state as the following chemical reaction processes or contact processes: *S* + *I* → 2*S*(*α*) and *I* → *R*(*β*), where *α* and *β* represent reaction rates.

There are remarkable analogies between epidemics and rumor diffusion, and many studies on rumor diffusion have employed the SIR model [17–19]. In the case of epidemics, viruses spread by physical transmission, such as through coughing; on the other hand, vicious rumors spread by virtual contact over the Internet. After the transmission, some people become infected in the case of epidemics and, similarly, some people believe the rumor (become infected with a vicious rumor) and spread the rumor through their tweets, causing rumor spread. After the infection, people recover over time, while in the case of rumors, people become tired of communicating or have the accurate information, and stop tweeting the rumor. We may regard public announcements as corresponding to vaccines in the case of epidemics and, therefore, many people move from a susceptible state to a recovered state. It is not easy to judge when a patient has recovered in the case of epidemics; however, in the case of rumors, we can precisely observe the alteration of users’ state from an infected state to a recovered state through data analysis.

**Fig 3:**
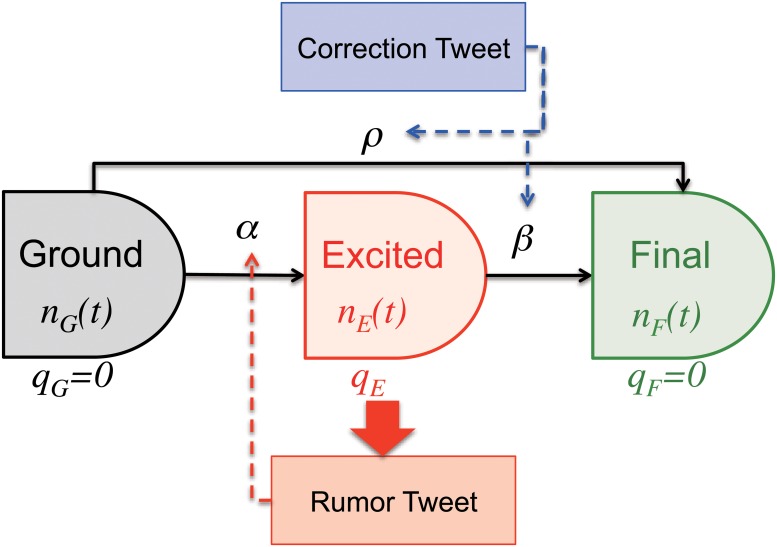
State transition diagram of our model with transition probabilities per minute *α*, *β*, and *ρ*. Users tweet the rumor depending on their state (Ground, Excited, and Final) with probability *q*_*E*_, and *q*_*G*_ = *q*_*F*_ = 0, respectively. Total number of users is *n* = *n*_*G*_(*t*) + *n*_*E*_(*t*) + *n*_*E*_(*t*). *α* changes with the number of rumors, and *β* and *ρ* change with the number of rumor-corrections as given by Equation (2), (3), and (4).

Since our model is inspired by traditional SIR model, we employ the following three states for users [32], as shown in Fig. 3.

- Ground state (*G*): users who have not encountered the rumor
- Excited state (*E*): users who believe the rumor
- Final state (*F*): users who already know that the rumor is false

Users tweet a rumor stochastically, depending on their state. At first, most users are in the Ground state. Upon receiving a rumor tweet, the user has a probability *α* of becoming infected, in which case he/she enters the Excited state. In the Excited state, he/she has a high probability, *q*_*E*_, of submitting a rumor tweet. When the user receives a correction tweet, he/she moves to the Final state with probability *β*. A user can go directly from the Ground to the Final state with probability *ρ* (Fig. 3). We saw many examples of users who only submitted a correction tweet, with no history of originally submitting the rumor. We assume there are *n* users in total, divided between the Ground state (*n*_*G*_(*t*)), Excited state (*n*_*E*_(*t*)), and Final state (*n*_*F*_(*t*)). Our initial condition is *n*_*G*_(0) = *n*, *n*_*E*_(0) = 0, and *n*_*F*_(0) = 0.

In order to run simulations, we set the seven parameters (*r*, *q*_*G*_, *q*_*E*_, *q*_*F*_, *α*, *β*, *ρ*). We systematically estimated these parameters, as shown in Table 1, to minimize the error between real data and simulation results. First, we determine the tweet probability *r* from real data. We define the active time of each user as the time range from the first tweet to the last tweet. From the active time, we can calculate the number of active users *U*(*t*) as the number of users who are active at time *t*. We calculate the tweet probability from the number of active users and the number of whole tweets *W*(*t*) at time *t*.

$$\begin{array}{c} r=\left\langle \frac{W(t)}{U(t)}\right\rangle \approx 0.01. \end{array}$$ (1)

Second, we set the probability *q*_*G*_ and *q*_*F*_ as zero because users in the Ground state do not know the rumor, and users in the Final state have lost interest in the rumor or they notice that the rumor is false.

**Table 1:** Detailed values of simulation parameters in Fig. 6(a). Tweet density of rumors *R*(*t*) and rumor-corrections *R*_*c*_(*t*) are introduced. Initial number in Excited state is *n*_*E*_(0) = 0.

	**Values**
*n*	10^6^
*r*	0.010
*q*_*G*_	0
*q*_*E*_	3.3 × 10^−2^ (*t* = 1289)
	2.9 × 10^−2^ (1840 ≤ *t* ≤ 1950)
	2.1 × 10^−2^ (1970 ≤ *t* ≤ 2160)
	2.5 × 10^−2^ (*otherwise*)
*q*_*F*_	0
*α*	3.5 × 10^−2^ (*t* = 1140)
	0.35*R*(*t*) (1141 ≤ *t* ≤ 1340, 1423 ≤ *t* ≤ 2160)
	0.35*R*(*t*) + 0.12 (*t* = 1289)
	0.35*R*(*t*) + 1.0 × 10^−2^ (1740 ≤ *t* ≤ 1750)
	0.35*R*(*t*) + 0.15 (*t* = 1765)
	0.79*R*(*t*) (1341 ≤ *t* ≤ 1422)
	1.1*R*(*t*) (2161 ≤ *t* ≤ 3240)
*β*	3.2 × 10^−2^ (1290 ≤ *t* ≤ 1340)
	5.9 × 10^−3^ (1341 ≤ *t* ≤ 1739)
	6.5 × 10^−2^ (1740 ≤ *t* ≤ 1790)
	0.30*R*_*c*_(*t*) + 3.5 × 10^−3^ (2161 ≤ *t* ≤ 2475)
	0.30*R*_*c*_(*t*) + 9.5 × 10^−3^ (2476 ≤ *t* ≤ 3240)
	3.5 × 10^−3^ (*otherwise*)
*ρ*	0.80*R*_*c*_(*t*) (2161 ≤ *t* ≤ 3240)
	0 (*otherwise*)

Finally, we choose the other four parameters (*α*, *β*, *ρ*, *q*_*E*_). We assume that these parameters are determined by the following representations:

$$\begin{array}{ccc} \alpha & = & k_{\alpha }^{\left(1\right)}R\left(t\right)+k_{\alpha }^{\left(2\right)} \end{array}$$ (2)

$$\begin{array}{ccc} \beta & = & k_{\beta }^{\left(1\right)}R_{c}\left(t\right)+k_{\beta }^{\left(2\right)} \end{array}$$ (3)

$$\begin{array}{ccc} \rho & = & k_{\rho }R_{c}\left(t\right) \end{array}$$ (4)

$$\begin{array}{ccc} q_{E} & = & k_{q_{E}} \end{array}$$ (5)

where *R*(*t*) is rumor density and *R*_*c*_(*t*) is rumor-correction density. We choose these six parameters, $\left(k_{\alpha }^{\left(1\right)},k_{\alpha }^{\left(2\right)},k_{\beta }^{\left(1\right)},k_{\beta }^{\left(2\right)},k_{\rho },k_{q_{E}}\right)$ to run simulations. In this procedure, we use the rumor density and individual dynamics.

For rumor density, we define the real time series *R*_*r*_(*t*) as follows.

$$\begin{array}{c} R_{r}\left(t\right)=\frac{w(t)}{W(t)} \end{array}$$ (6)

where *w*(*t*) is the number of rumor tweets at time *t*. We also define the simulated time series *R*_*s*_(*t*), which we can calculate using the model parameters as follows. We define random variables *S*_*i*_(*t*) and $Q_{i}^{\left(\sigma \right)}(t)$ for each user *i* with state *σ*_*i*_(*t*) ∈ {*G*, *E*, *F*} by using uniform random numbers *x*_*i*,*t*_, *y*_*i*,*t*_ ∈ [0, 1).

$$\begin{array}{ccc} S_{i}\left(t\right) & = & \left\{\begin{array}{cc} 1 & x_{i,t}<r\\ 0 & otherwise \end{array}\right. \end{array}$$ (7)

$$\begin{array}{ccc} Q_{i}^{\left(\sigma \right)}\left(t\right) & = & \left\{\begin{array}{cc} 1 & y_{i,t}<q_{\sigma }\\ 0 & otherwise \end{array}\right. \end{array}$$ (8)

From these variables, we can define *R*_*s*_(*t*) as follows.

$$\begin{array}{c} R_{s}\left(t\right)=\frac{\sum_{i:\sigma_{i}\left(t\right)\in G}S_{i}\left(t\right)Q_{i}^{\left(G\right)}\left(t\right)+\sum_{i:\sigma_{i}\left(t\right)\in E}S_{i}\left(t\right)Q_{i}^{\left(E\right)}\left(t\right)+\sum_{i:\sigma_{i}\left(t\right)\in F}S_{i}\left(t\right)Q_{i}^{\left(F\right)}\left(t\right)}{\sum_{i}S_{i}\left(t\right)} \end{array}$$ (9)

The average behavior of *R*_*s*_(*t*) of many trials is given as follows, with the condition that *q*_*G*_ = *q*_*F*_ = 0.

$$\begin{array}{c} \left\langle R_{s}\left(t\right)\right\rangle =\frac{n_{G}\left(t\right)rq_{G}+n_{E}\left(t\right)rq_{E}+n_{F}\left(t\right)rq_{F}}{nr}=\frac{n_{E}\left(t\right)q_{E}}{n} \end{array}$$ (10)

For individual dynamics, we focus on the first posting line (FPL), which corresponds to the normalized accumulated number of users who tweet rumors. We define real FPL *F*_*r*_(*t*) as follows.

$$\begin{array}{c} F_{r}\left(t\right)=\sum_{t=t_{\text{min}}}^{t_{\text{max}}}\frac{N(t)}{W(t)} \end{array}$$ (11)

Here, *N*(*t*) is the number of users who tweet a rumor for the first time at time *t*. We also define simulated FPL *F*_*s*_(*t*). We simulate twenty times for each parameter, and calculate *R*_*s*_(*t*) and *F*_*s*_(*t*) as the median value.

Next, we define errors *ϵ*_1_ and *ϵ*_2_ as follows.

$$\begin{array}{ccc} \epsilon_{1} & = & \sum_{t=t_{\text{min}}}^{t_{\text{max}}}{\left(\frac{R_{r}\left(t\right)-R_{s}\left(t\right)}{\left\langle R_{r}\right\rangle }\right)}^{2} \end{array}$$ (12)

$$\begin{array}{ccc} \epsilon_{2} & = & \sum_{t=t_{\text{min}}}^{t_{\text{max}}}{\left(\frac{F_{r}\left(t\right)-F_{s}\left(t\right)}{F_{r}\left(t\right)}\right)}^{2} \end{array}$$ (13)

*ϵ*_1_ and *ϵ*_2_ are the square of the relative error between the real and simulated time series. However, we use the average of time series ⟨*R*_*r*_⟩ instead of *R*_*r*_(*t*) in the denominator of Equation (12) to avoid overestimation caused by the case that the denominator *R*_*r*_(*t*) has a small value.

**Fig 4:**
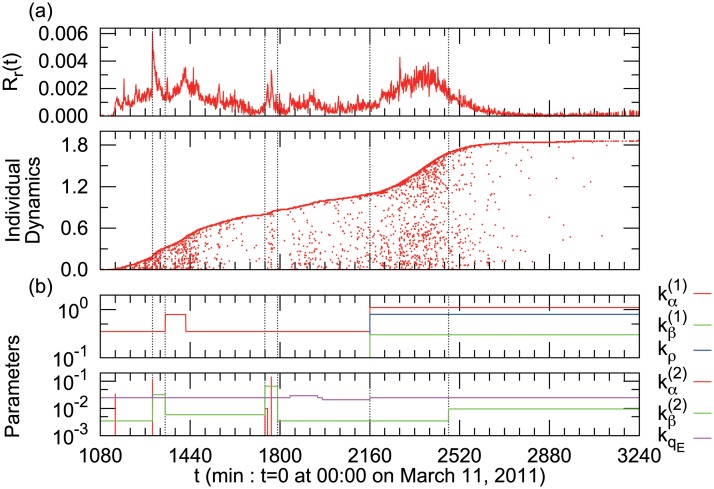
(a) Segmentation of the time series and estimated parameters. Vertical dashed lines represent the boundaries of the segments in the simulation. These boundaries are determined manually by changes in the tweet density and curvature of individual dynamics. **(b)Time series of parameter values in the simulation shown in Fig. 6(a).** (Top) Coefficients of time-varying terms in Equation (2), (3), and (4). (Bottom) Coefficients of time-invariant terms in Equation (2), (3) and (5).

Depending on the clear peaks and the trends in the empirical result of rumor spreading as shown in Fig. 4, we divide the time series into seven parts manually, and estimate the parameters in each time window to minimize the errors. We assume that there are two causes for the peaks of rumor density: one is the news of the gas tank explosion (*t* = 1289), and the other is the hub user who has many connections with users and a great spreading force (*t* = 1765).

**Fig 5:**
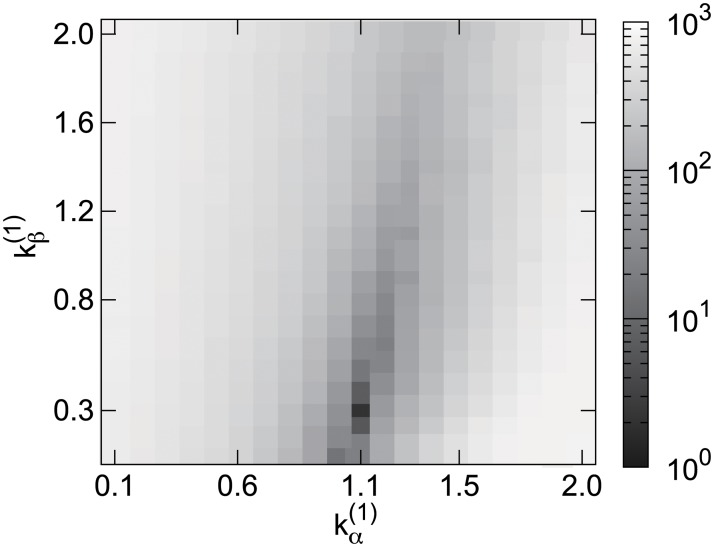
Heat map of optimal parameters in *t* ∈ [2161, 2475] shaded by *Rank*(*ϵ*_1_) + *Rank*(*ϵ*_2_), Equation (14). Horizontal axis is $k_{\alpha }^{\left(1\right)}$ in Equation (3) and vertical axis is $k_{\beta }^{\left(1\right)}$ in Equation (2) which both vary with time.

However, the order is different between *ϵ*_1_ and *ϵ*_2_; therefore, we define *Rank*(*ϵ*_1_) and *Rank*(*ϵ*_2_) as rank in ascending order of each parameter sets. In addition, we define the optimal parameter set $\left(k_{\alpha }^{(1)*},k_{\alpha }^{(2)*},k_{\beta }^{(1)*},k_{\beta }^{(2)*},k_{\rho }^{*},k_{q_{E}}^{*}\right)$ as follows.

$$\begin{array}{c} \left(k_{\alpha }^{(1)*},k_{\alpha }^{(2)*},k_{\beta }^{(1)*},k_{\beta }^{(2)*},k_{\rho }^{*},k_{q_{E}}^{*}\right)=\underset{\left(k_{\alpha }^{\left(1\right)},k_{\alpha }^{\left(2\right)},k_{\beta }^{\left(1\right)},k_{\beta }^{\left(2\right)},k_{\rho },k_{q_{E}}\right)}{\arg\;\min}\left[Rank\left(\epsilon_{1}\right)+Rank\left(\epsilon_{2}\right)\right] \end{array}$$ (14)

where “arg min” is the argument for the minimization of *Rank*(*ϵ*_1_) + *Rank*(*ϵ*_2_). Fig. 5 shows an example of the heat map of the sum of the ranking with the parameters $k_{\alpha }^{\left(1\right)}$ and $k_{\beta }^{\left(1\right)}$ in *t* ∈ [2161, 2475]. The other parameters do not appear in Fig. 5, but they are optimized in Equation (14) with $\left(k_{\alpha }^{(2)*},k_{\beta }^{(2)*},k_{\rho }^{*},k_{q_{E}}^{*}\right)=(0,3.5\times 10^{-3},0.80,2.5\times 10^{-2})$. In this case, we choose the parameters $k_{\alpha }^{(1)*}=1.1$ and $k_{\beta }^{(1)*}=0.30$ from this heat map.

**Fig 6:**
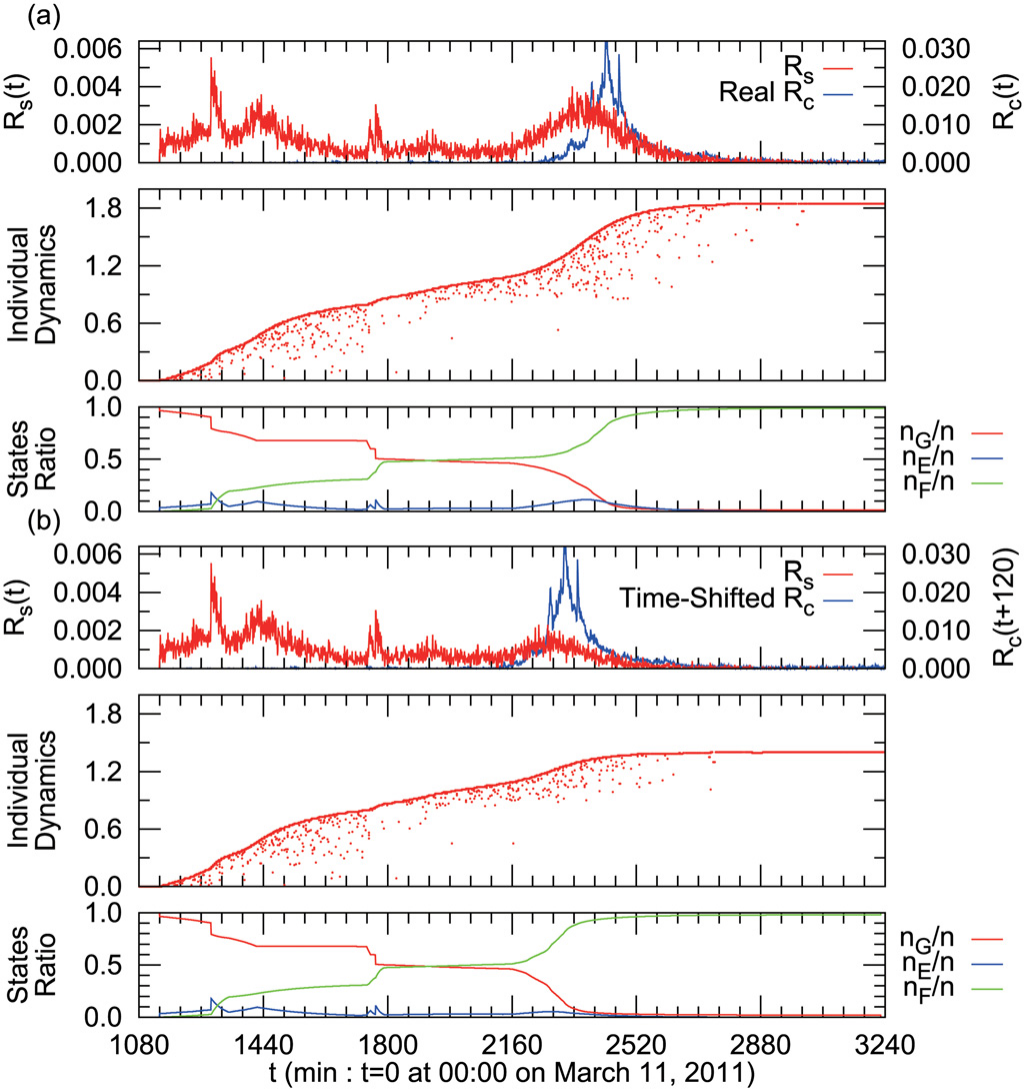
(a) Simulation of rumor diffusion reproducing the empirical result of Fig. 1. **(b) Simulation of rumor diffusion if correction tweets had appeared 120 minutes earlier.** By comparison of (a) and (b), the Rumor Density *R*(*t*) around *t* = 2,400 is almost diminished and the final number of rumor tweeted users decreased as shown in Individual Dynamics.

The top chart in Fig. 6(a) is the simulated rumor density, *R*_*s*_(*t*), and the second chart represents the simulated individual dynamics of users’ tweets. Both charts are close to the real data shown in Fig. 1, by choosing appropriate parameters. The third chart is the dynamics of portions with each state. From the values of parameters, we can estimate the probability of submitting a tweet in each state (*q*_*G*_, *q*_*E*_, *q*_*F*_), the probability of a transition to another state (*α*, *β*, *ρ*) in Fig. 4(b) and Table 1, and the number of users in each state (*n*_*G*_(*t*), *n*_*E*_(*t*), *n*_*F*_(*t*)) in Fig. 6(a), which are not directly observable.

Using this model, we can simulate various situations of rumor diffusion. Fig. 6(b) shows a simulation in which the official announcement of City Hall is declared two hours earlier than the actual data. The peak around *t* = 2300 becomes clearly smaller than the actual case of Fig. 6(a). This result demonstrates that this early announcement can work as a preventive effect and reduces the rumor spread.

## Discussion

Most rumors are not serious, but sometimes rumors can cause significant confusion and result in time and economic losses. While conventional rumors are difficult to observe, and their diffusion and convergence take a long time, rumors that are spread electronically (e.g., via Twitter) are precisely observable, and the characteristic time scale is much shorter, as we have seen here.

With regard to the rumor convergence, we found that the official announcement played a key role. Here, 37.3% of users who received a rumor-correction tweet from City Hall directly retweeted the message. On the other hand, the most retweeted user was only connected to 5.4% of the users who received the rumor tweet. There are several pieces of evidence that an official announcement can stop the rumors across the world, because governments aggressively intervene to correct rumors [13, 29, 30]. Our result regarding rumor convergence supports this evidence that the official announcement can play a key role in stopping rumors.

The agent-based stochastic model of rumors which is inspired by SIR model helps us in understanding the complicated social phenomenon of when rumors spread. As we have shown, the model can reproduce the actual time evolution of rumors. Through a process of parameter fitting using the real data, we can estimate quantities such as “the infection rate of the rumor” and “the ratio of users who still believe the rumor,” which cannot be observed without the model. In addition, we can repeat numerical experiments to establish how to suppress rumor growth. For example, we show that the total number of rumor tweets would have been much smaller had the City Hall issued a correction tweet earlier than it did.

There are four tasks to enhance the applicability of the present model. First, we should extend our analysis to languages other than Japanese to clarify language dependence. Second, we need to introduce fully automatic categorization of tweets into rumors and rumor-corrections, as there were cases in which our separation algorithm required human judgment, which was quite time consuming. Third, the data assimilation method can be revised to estimate more accurate time-dependent values with a lower calculation cost. At present, we simply calculate the square of relative errors. Finally, in this paper, we proposed our model from the view point of minimal modeling, and our model satisfies all major properties of rumor diffusion and conversion observed in the real data. Other types of modeling for the rumor diffusion and conversion may also be possible. Comparison between other types of model such as Lotka-Volterra predator-prey model of ecosystem and our model is also an interesting problem.

Rumor diffusion is a universal phenomenon, can occur anywhere in the world, and has throughout history. Here we study only one empirical case of rumor diffusion, but we believe it is important to discover the universal properties of rumors beyond the borders of languages and nationalities. As we now have tools to observe rumors in cyberspace, it will become possible to detect malignant rumors automatically at an early stage in real time.

## Materials and Methods

### Data description

**Fig 7:**
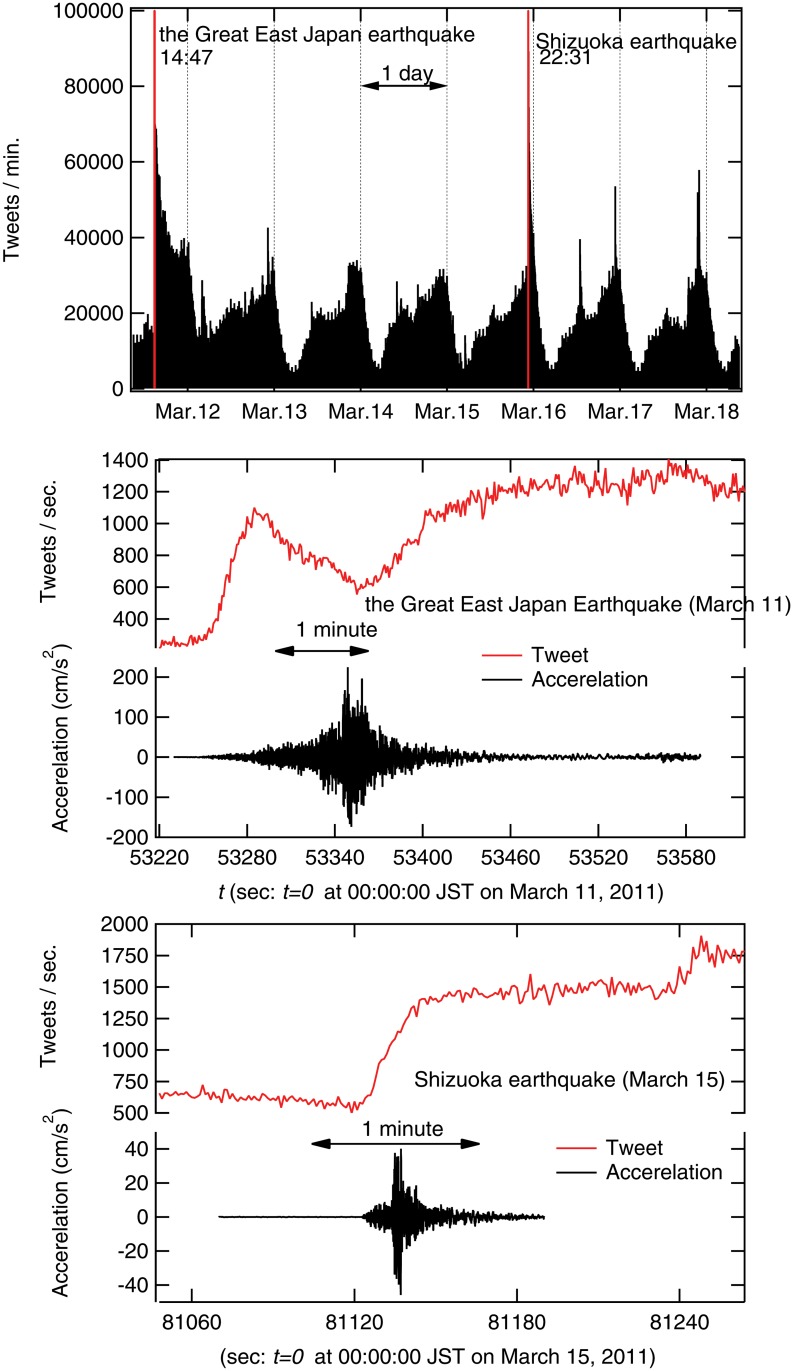
Number of whole tweets per minute from March 11 to 17 in 2011 (top). **Number of tweets per second with ground acceleration at the very moment of the quake on March 11 (middle) and 15 (bottom).** Red colored areas in the top figure are enlarged in the lower figures, which depict the very moment of the quakes. There are two big earthquakes on March 11 and 15 in 2011. One is the Great East Japan earthquake that registered 9.0 on the Richter scale in the Tohoku area on March 11, and the other hit Shizuoka area (middle-eastern Japan) on March 15, with 6.4 on the Richter scale. The ground acceleration data are obtained from the Japan Meteorological Agency (http://www.seisvol.kishou.go.jp/eq/kyoshin/jishin/index.html).

**Fig 8:**
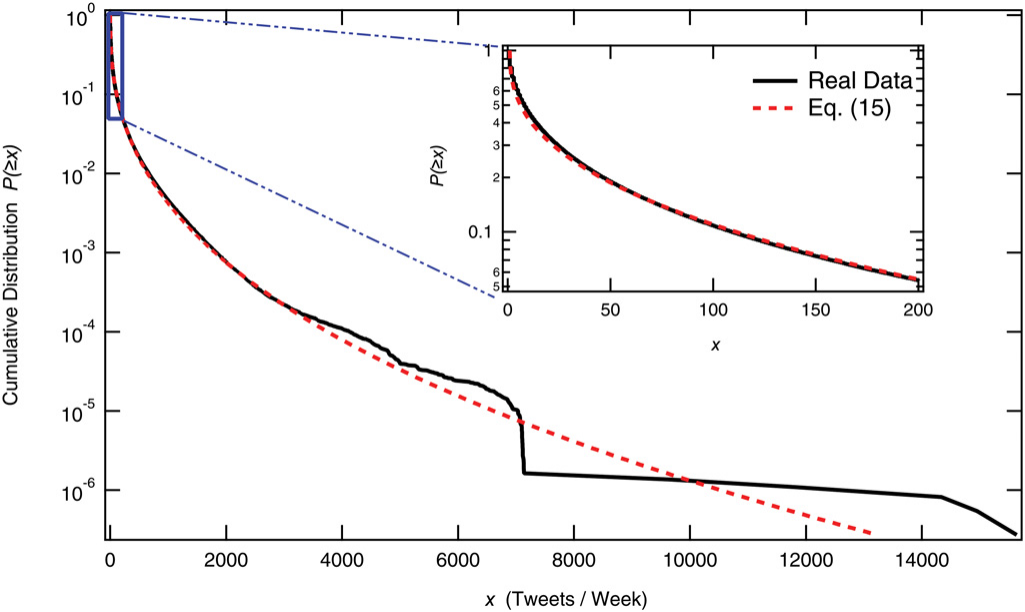
Cumulative distribution of number of tweets per user during one week after the quake *x* in a semi-log plot. Red dashed lines is the stretched exponential function introduced in Equation (15). (Inset) An enlarged part of the same figure in *x* ∈ [1, 200]. The most tweeted user is known as a “bot” who tweeted 15,635 times during the week. The bot is a kind of robot that automatically tweets information about the missing person’s safety with the hash tag “#pf_anpi.” The median value of tweets per user during the week is seven, that is, one tweet per day.

We analyzed all Japanese tweets from 09:00, March 11, 2011, to 09:00, March 17, 2011. In total, these included 179,286,297 tweets from 3,691,599 users (Fig. 7). In Fig. 8, cumulative distribution of tweets per users is plotted in semi-log scale. The median of the tweets during this week is seven tweets per user while the mean is 48. The most tweeted users are “bots” that automatically generate tweets about missing people. The bots repeated their announcement and tweeted more than 150 thousand times during the week. Here, we found that a stretched exponential function is well-fitted as follows.

$$\begin{array}{c} P\left(\geq x\right)=\exp\left[\frac{-\lambda {\left(x-1\right)}^{\gamma }}{\tau }\right] \end{array}$$ (15)

where *λ* = 1.0, *γ* = 0.39, and *τ* = 2.9 that are estimated in the range *x* ∈ [1, 2800] by Levenberg-Marquardt algorithm [33].

The data format for each tweet consists of the following tags: (1) tweet ID (simple sequential number); (2) user ID (anonymously assigned number); (3) time stamp in seconds; and (4) the main text of the tweet, limited to 140 Japanese characters. Official data were provided by Twitter Japan, Inc. through Project 311 [34]. The project was organized by Google Japan, and several companies gave support by providing data, including broadcasted TV programs, newspaper articles, and GPS information from mobile phones and automobiles. The aim of the project was to extract insights that might be useful in the event of other disasters by attracting researchers who were interested in analyzing large amounts of data from this confusing period. The project was held in 2012 for two months with the final session of the participants. During the period, the participants can use the data under the agreement with Twitter Japan Inc. The same data we analyzed in our paper can be purchased from Twitter for the purpose of scientific study (https://twitter.com/).

### Identify rumor and correction

We identified those rumor tweets that included false information by choosing appropriate keywords. More than 30 rumors from different sources were confirmed during the week under observation. An example of a rumor was “For prevention of side effects from radioactivity, it is good to drink mouthwash including iodine and to eat as much seaweed as you can.” This information was rapidly revealed to be false. We then chose the largest of these rumors.

Since rumor tweets often appear simultaneously with rumor-correction tweets, distinguishing rumors from rumor corrections is not a straightforward process. To avoid possible misleading tweets, we randomly selected and read approximately 10,000 tweets to differentiate between rumors and corrections. We carefully extracted rumor and rumor-correction tweets by combining logical conjunctions and negations.

**Fig 9:**
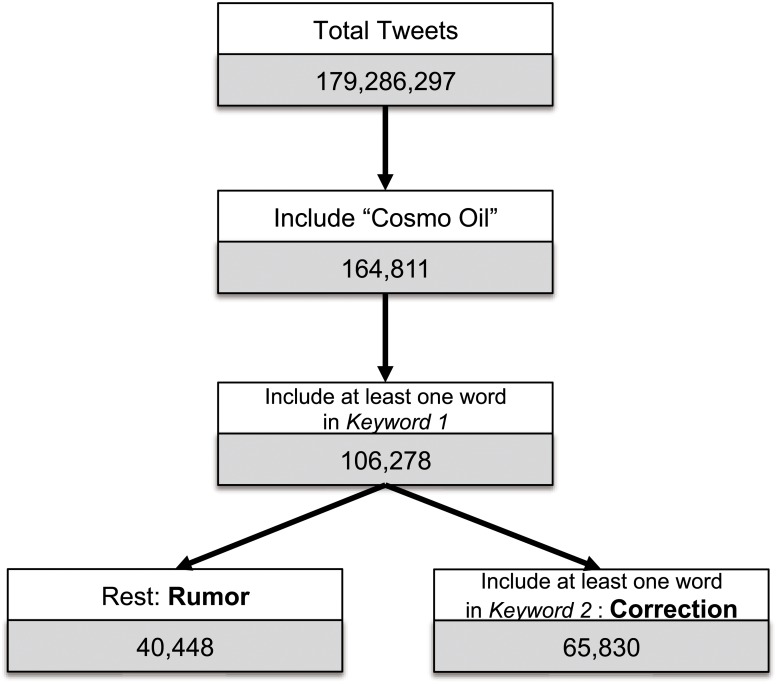
Flow diagram of the tweet detection algorithm for rumor and correction. By combining several keywords listed in Table 2 and 3, rumor and rumor-correction tweets are extracted.

First, we selected tweets including the word “cosmo oil (コスモ石油),” the name of the oil company that had the accident, yielding 164,811 tweets. Among these tweets, we identified rumor-related tweets that included one of four keywords as listed in Table 2 giving us 106,278 tweets. We then separated these remaining tweets into rumor and rumor-correction tweets by searching for the keywords listed in Table 3. To divide the tweets, we identified a tweet as a rumor-correction tweet if it contained more than one keyword relating to rumor correction, as shown in Table 3. The remainder of the tweets were identified as rumor tweets (Fig. 9).

**Table 2:** *Keyword 1* that used for identifying rumor-related tweets as shown Fig. 9.

**Keyword (English)**	**Keywords (Original form)**
umbrella	傘
mac	カッパ
harmful material	有害物質
raincoat	レインコート

**Table 3:** *Keyword 2* for identifying correction tweets as shown Fig. 9.

**Keyword (English)**	**Keywords (Original form)**
correction	訂正
error	デマ, デマ, デ マ, [single/double space inserted]
bum steer	ガセ
misinformation	誤情報
contradiction	否定
fallaciousness	虚偽
chain mail	チェーンメール, チェーンメイル [different character expression]
no fact at all	事実はありません
harmlessness	無害
said no (without “said dangerous”)	ないそうです [without 危ないそうです]
lie	嘘, ウソ [Chinese/Katakana character expression]
no basis in fact	事実無根
without the occurrence	発生しない
mistake (without “no mistake”)	間違 [without 間違いない]
LP gas	LP ガス
false report	誤報

We identify a correction when a tweet contains at least one of the following keywords.

Finally we had 40,448 rumors from 38,226 users and 65,830 rumor-corrections from 56,818 users for the analysis. Our algorithm has 98% accuracy for rumor and rumor-correction tweets, which we established by checking 100 random tweets. The misleading tweets were caused by tweets that were difficult to distinguish between rumor and rumor-correction. (Some examples of the rumor, correction and mis-categorized tweets are listed in S1 Text. Only 15.7% of users who tweeted rumors sent rumor-correction tweets. There may have been users who had sent rumor tweets who just deleted the tweet when they have noticed the wrong information. However, we cannot trace these activities from this data.

## Supporting Information

S1 Text Examples of rumor, rumor-correction and mis-categorized tweets. This document contains the tweet examples. The original tweets are Japanese, but they are translated into English by the authors.
(PDF)
