# Supplementary material for: Rumor Diffusion and Convergence during the 3.11 Earthquake: A Twitter Case Study
Source: PLoS One. 2015 Apr 1;10(4):e0121443. doi: 10.1371/journal.pone.0121443 (PMC4382198; doi:10.1371/journal.pone.0121443)
Supplement: S1 Text — This document contains the tweet examples. The original tweets are Japanese, but they are translated into English by the authors. (PDF) [file pone.0121443.s001.pdf]

Text S1: Examples of tweets

An example of rumor

[PLEASE SPREAD THE NEWS] I forward an email from a factory worker nearby Cosmo Oil in Ichihara, Chiba. Caution when going out! DO NOT expose your skin! Harmful materials caused by the Cosmo Oil explosion are attached to the clouds. They will fall on the ground when it rains. Bring your umbrella and raincoat to protect your skin! They will move roughly 100km when they are attached to the clouds.

An example of rumor (Retweeted)

RT @(a user name): [PLEASE SPREAD THE NEWS] People who live near Chiba area! Harmful materials caused by the Cosmo Oil explosion are attached to the clouds and falls with rains. So please bring your umbrella and raincoat when you go out to prevent your skin from getting in contact with rains.

An example of correction

Although the Cosmo Oil tank explosion is true, there is no evidence that there are harmful materials attached to the clouds and that they will fall with rains. DO NOT spread wrong or uncertain information. http://(URL)

An example of correction (Retweeted)

RT @(the city hall account): There is a chain mail with wrong information for residences in Chiba and in the neighborhood area which state that the harmful materials will scatter with rains because of the Cosmo Oil LPG tank explosion in Ichihara city. We confirmed with the earthquake disaster prevention section in Chiba Fire Department that it is NOT TRUE.

An example of mis-categorized tweet (actually correction, but categorized into rumor)

There seems to be emails about harmful materials caused by Cosmo Oil. However, there are no official announcements from Chiba City website and Ministry of Health, Labor and Welfare website at 14:35 present. The Cosmo Oil website is hardly accessible. Whether true or false, the lack of official announcements could lead to confusion.

An example of mis-categorized tweet (actually correction, but categorized into rumor)

Do not expose your skin, be careful of rains which contains harmful materials... I cannot stop laughing! how stupid! who is deceived by such chain-mail?

An example of mis-categorized tweet (actually rumor, but categorized into correction)

Regarding the post about the rain with harmful materials caused by the Cosmo Oil explosion, I wonder if it is true because no one says it is misinformation at all... I am a little afraid.

An example of mis-categorized tweet (actually rumor, but categorized into correction)

Is the RT spread about rain with harmful materials caused by Cosmo Oil explosion true? I cannot say it is false because it may happen, but I hardly believe in it.
